# Extension modules for storage, visualization and querying of genomic, genetic and breeding data in Tripal databases

**DOI:** 10.1093/database/bax092

**Published:** 2017-12-09

**Authors:** Sook Jung, Taein Lee, Chun-Huai Cheng, Stephen Ficklin, Jing Yu, Jodi Humann, Dorrie Main

**Affiliations:** Department of Horticulture, Washington State University, Pullman, WA, 99164, USA

## Abstract

Tripal is an open-source database platform primarily used for development of genomic, genetic and breeding databases. We report here on the release of the Chado Loader, Chado Data Display and Chado Search modules to extend the functionality of the core Tripal modules. These new extension modules provide additional tools for (1) data loading, (2) customized visualization and (3) advanced search functions for supported data types such as organism, marker, QTL/Mendelian Trait Loci, germplasm, map, project, phenotype, genotype and their respective metadata. The Chado Loader module provides data collection templates in Excel with defined metadata and data loaders with front end forms. The Chado Data Display module contains tools to visualize each data type and the metadata which can be used as is or customized as desired. The Chado Search module provides search and download functionality for the supported data types. Also included are the tools to visualize map and species summary. The use of materialized views in the Chado Search module enables better performance as well as flexibility of data modeling in Chado, allowing existing Tripal databases with different metadata types to utilize the module. These Tripal Extension modules are implemented in the Genome Database for *Rosaceae* (rosaceae.org), CottonGen (cottongen.org), Citrus Genome Database (citrusgenomedb.org), Genome Database for Vaccinium (vaccinium.org) and the Cool Season Food Legume Database (coolseasonfoodlegume.org).

**Database URL**: https://www.citrusgenomedb.org/, https://www.coolseasonfoodlegume.org/, https://www.cottongen.org/, https://www.rosaceae.org/, https://www.vaccinium.org/

## Introduction

Tripal (1, 2), a toolkit for construction of online biological databases uses two open source tools, the Chado database schema (3) and Drupal (https://www.drupal.org/), an open source Content Management Systems (CMS). The unprecedented volume of large-scale data being generated for non-model species, has led to an increasing need for online community databases where these data can be stored, integrated, visualized and made available for further analyses in such a way that fits each community. Building biological databases is a non-trivial task, requiring extensive skills and time from experienced programmers and close collaboration with biological curators or scientists. Tripal significantly reduces the time and cost for database construction and management resulting in its increasing adoption by many communities. Some example databases using Tripal include GeneNetEngine (4), the Banana Genome Hub (http://banana-genome.cirad.fr/) (5), CottonGen (https://www.cottongen.org/) (6), the Genome Database for *Rosaceae* (GDR, http://www.rosaceae.org) (7), Knowpulse: Pulse Crop Genomics & Breeding (http://knowpulse2.usask.ca/portal) (8), the Hardwood Genomics Database (http://www.hardwoodgenomics.org/) (9), the i5k Workspace (https://i5k.nal.usda.gov/) (10), the Cool Season Food Legume Database (CSFL, https://www.coolseasonfoodlegume.org) (11), the Legume Information System (http://legumeinfo.org/) (12) and the Citrus Genome Database (CGD, http://www.citrusgenomedb.org) (13). For a more extensive list of databases currently using Tripal see https://tripal.info.

Drupal, an open source, popular and well-supported CMS, simplifies web site installation, web site development and content management and has been used to construct a wide variety of websites and applications. Drupal provides security, performance, account management and is extensible via an Application Programming Interface (API) that allows site developers to create new PHP modules. Drupal has one of the largest open-source communities in the world and maintains a repository of thousands of user-contributed modules and themes.

Tripal is a suite of Drupal modules which allows management and display of biological data stored in the Chado database. Chado is a database schema and a member of GMOD, the Generic Model Organism Database project (www.gmod.org), a collection of open source software tools for managing, visualizing, storing and disseminating genetic and genomic data. Tripal also provides an API for creation of custom functionality. As developers of community-databases require new functionality, they can create new Tripal compatible extension modules using both the Drupal and Tripal APIs. Tripal offers a list of many of these user-contributed extension modules on the Tripal website (www.tripal.info).

Chado is an open-source, community-derived database schema for PostgreSQL. It was originally developed by FlyBase (14) to house Drosophila data that integrates annotated genomic sequences, genetic, phenotypic and bibliographic data. The schema was developed to be generic, modular, ontology-driven and open source so that it can be used as common data store for databases and tools that need to store data for other organisms. Chado extensively uses ontologies and controlled vocabularies to describe data and their relationships. For example, features on a genome are described using the Sequence Ontology (SO) (15), such as ‘gene’ and ‘QTL.’ The relationship between different genomic features are described by terms such as ‘is_a’ and ‘located_in’ which are also present in the SO. The Relations Ontology (http://www.obofoundry.org/ontology/ro.html) contains terms for creating relationships between a varieties of data. One important characteristic of Chado is its modular design. Chado tables are organized into groups, called modules, such as sequence, genetic, phenotype, map, stock, organism, library, expression, controlled vocabulary and general modules. Each module represents distinct domains of data. This allows new modules to be added when sufficient need arises. In 2011, a Natural Diversity module was added to Chado through collaborative efforts by a consortium of representatives from several online genome database projects (16) to support data from multiple large-scale phenotypic and genotypic projects.

The ontology-driven design of Chado allows database developers to store data for new biological concepts, and new experimental techniques without constantly changing the schema. The adoption of Chado, however, involves a steep learning curve due to this general and ontology-driven design. Best practices for storing genomic data are documented on the GMOD website (http://gmod.org/wiki/Chado_Best_Practices) as well as in the Chado manuscript (3) from FlyBase. Tripal has been mostly adopted by communities with newly generated large-scale genomic data and the documentation on how to store genetic, phenotypic and genotypic data along with genomic data has not been well documented except in a recent publication which describes case studies from GDR and CottonGen for storing genomic, genetic and breeding data (17). Despite these examples, it remains a necessity for site developers to program data loaders for data types such as markers, genetic maps, QTL/Mendelian Trait Loci (MTL), stocks, phenotypes and genotypes. Moreover, tools to display and search these data are also needed.

In response to these needs, a set of Tripal extension modules have been created: the Mainlab Chado Loader (MCL), the Mainlab Data Display and the Mainlab Chado Search module. These modules provide site developers with tools to collect and upload data, to organize and display data and to enable advanced search functions. Supported data types include organism, marker, QTL, MTL, germplasm, map, project, phenotype, genotype and their associated metadata. Even though the types of metadata stored can be different from one community to another, these modules will provide a good starting point to build a community database with the possibility of modification to suit each community’s need. The MCL provides a set of data templates in Excel format to collect data and respective loaders to import them into Chado. The Mainlab Chado Search module provides an accompanying set of search tools and the Mainlab Data Display module provides the appropriate online data pages. These Tripal extension modules, along with the Tripal core module, have been used in the construction of the GDR, CottonGen, CSFL, CGD and the Genome Database for Vaccinium (https://www.vaccinium.org/). These open-source Tripal extension modules with usage documentation can be found in the Tripal organization’s GitHub repository. They can also be accessed from http://tripal.info/extensions.

## Description

### Mainlab Chado Loader

**Figure 1.**
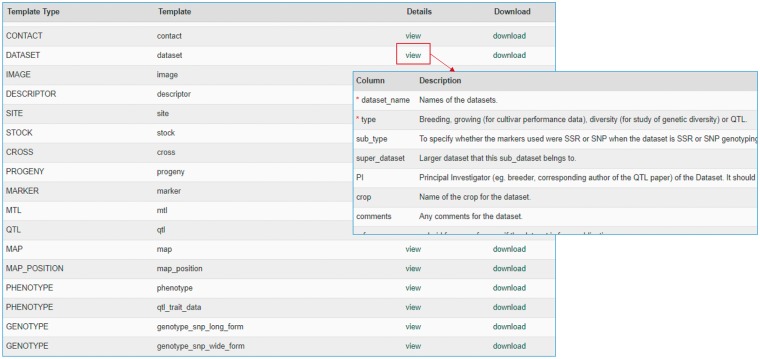
Data template page of the MCL user interface. Users can view the description of each data template or download the templates.

**Figure 2.**
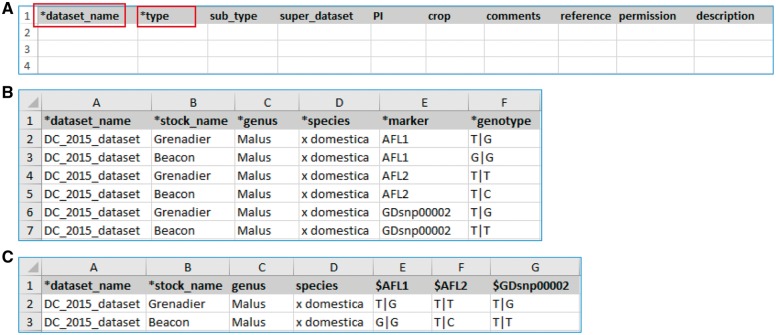
Example templates downloaded from the MCL module. (**A**) A template for contact data type. Columns with a prefix ‘*’are required fields. (**B**) A template for genotype datatype, ‘genotype_snp_long_form.’ (**C**) A template for genotype datatype, ‘genotype_snp_wide_form.’ Users can enter marker names as column headings with a prefix ‘$.’

The MCL module supports uploading of various biological data types into a Chado database. MCL provides both a user interface and an admin interface. The admin interface is for the site developer and the user interface is for data curators. The user interface is composed of pages for downloading data templates and data loading. Data templates are Excel files that contain metadata type as headings where data curators or researchers can enter data to be loaded into the database. In the current version of MCL, templates are available for each of the following data types: db (database), cv (controlled vocabulary), library, trait, contact, dataset, image, descriptor, site, stock, cross, progeny, marker, MTL, QTL, map, map position, phenotype and genotype. There are multiple templates for some data types. Templates for the same data type are defined to have the same template type. In the template page of the user interface, which can be accessed at https://yoursite.org/mcl/template_list once installed, users can view the description of each template and download the templates (Figure 1). The description of each template is available on the first sheet of each download file as well as on the template page of the user interface. As shown in Figure 2A, the column headings with * prefix in the templates are required fields. Figure 2B and C shows two templates that belong to the genotype template type, an example of template type that has multiple templates. In ‘genotype_snp_long_form’ (Figure 2B), there is a marker column where the marker used for genotyping is entered. Right next to it is a genotype column where the genotype is entered. So each row contains one stock name, one marker name and the genotype for the marker and stock combination. In this way, the number of the rows is the product of number of markers and number of stocks. For example, if 3 markers are used for 2 stocks, a total of 6 rows are entered. In ‘genotype_snp_wide_form’ (Figure 2C), marker names are entered as column headings with ‘$’ as a prefix. Each row contains one stock name and genotypes of all the markers used. In this way, the number of the rows is the same as the number of the stocks. For example, if 3 markers are used for 2 stocks, a total of 2 rows are entered. Having two different templates are convenient for users since the output of some genotyping software is similar to the wide form and others to the long form. When templates are needed for a new or existing template type or new metadata are needed for the existing template, a site developer can modify MCL to create a new template or columns for new metadata. The templates for the latest release of the module are also provided as a Supplementary Material.

**Figure 3.**
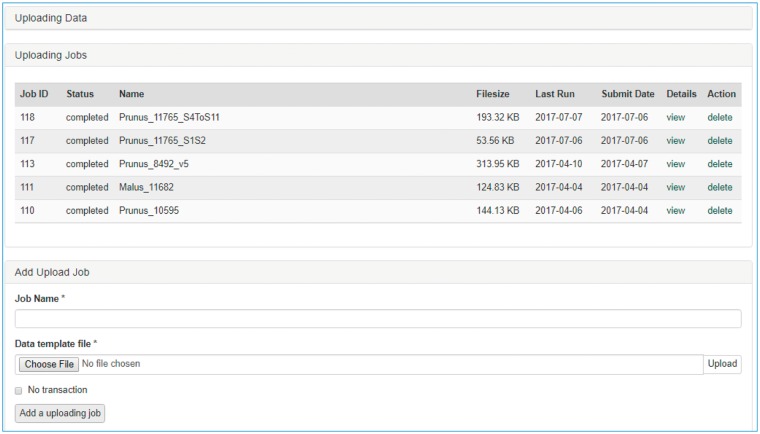
Data uploading page of the MCL user interface. The uploading page shows the status of all the submitted uploading jobs and provides links to each job details page.

**Figure 4.**
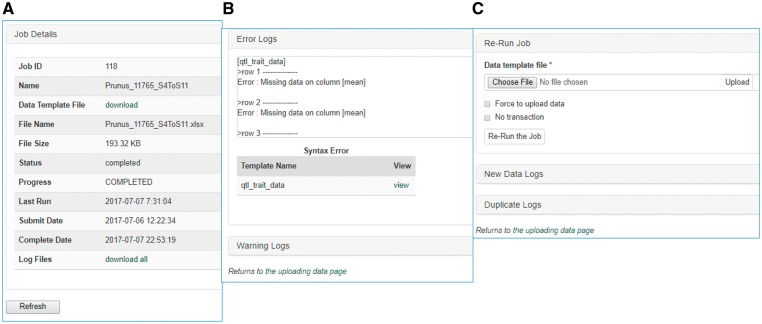
Sections of an uploading job detail page in MCL. (**A**) A table shows the details of the uploading job. (**B**) A window that shows error logs. (**C**) A section where users can re-run the job after fixing any errors. New data logs show any new data that have been uploaded and the duplicate logs show any data in the template that already exist in the database.

Once data is entered in the template, data curators can upload data through the web interface (Figure 3). The uploading page shows the status of all the submitted uploading jobs and provide link to a page where users can view the details of each uploading job (Figure 4). The MCL module loads data into Chado in three phases. First, data entry errors are checked. Examples of data entry errors include missing columns, missing data on required columns and misspelled column names. Second, data integrity is checked. For example, the marker names in the map_position template should already exist in Chado. If not, the loader returns an appropriate error message in the error log file. During the uploading phases, MCL creates several different log files such as error log file, new data log file and duplicate data log file which can be viewed in the job detail page (Figure 4). MCL terminates loading if it finds a data entry or integrity error and outputs an error log so the user can fix the data (Figure 4B). Users can re-run the job after fixing the data by submitting a corrected data file in the job detail page (Figure 4C). Finally, after data entry and integrity checks, the data in the template is loaded into Chado. A new data log file shows all the new data that has been loaded and the duplicate log file shows any data in the template that already exist in the database (Figure 4C). In addition to the web interface, a command-line interface is provided for site curators to automate loading via scripting if desired.

The admin interface is composed of five tabs: template type, template, user, variables and configuration. The template type tab allows the administrator to add a new template type and assign a rank. The rank specifies the order of loading to maintain referential integrity in Chado. The pre-specified rank allows data curators to load an Excel file with multiple data templates (e.g. contact, marker, map and map_position) without concerning the order of loading since MCL loads data based on the rank. The template tab allows administrators to choose the templates to be displayed in the user interface since not all the templates may be needed. In the user tab, an administrator can specify which users of the Drupal site can access the user interface for loading of templates. The variables tab allows the administrator to modify or add the site-wide controlled vocabularies that are used in the data templates. The configuration tab lets the administrator specify the MCL working directory and MCL library directory where files will be stored on the server during loading. Detailed instructions are available in the README document that accompanies the module. This module is available for download at https://github.com/tripal/mainlab_chado_loader/releases/latest.

### Mainlab Chado Search module

**Figure 5.**
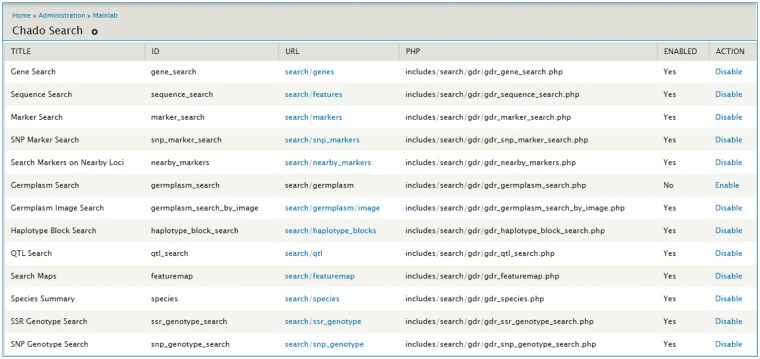
Mainlab Chado Search Admin page.

**Figure 6.**
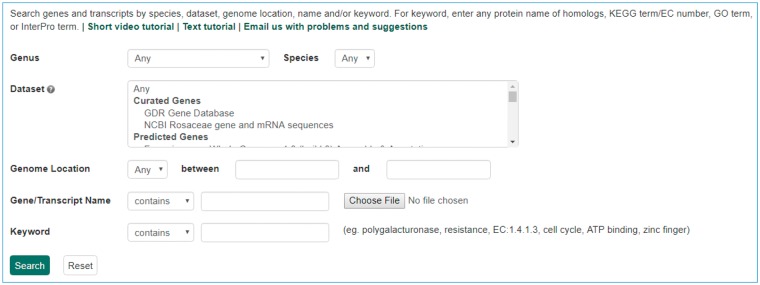
Search page for genes and transcripts in GDR. Users can search genes and transcripts using various filters such as genus, species, dataset, aligned genome location, name and keyword.

**Figure 7.**
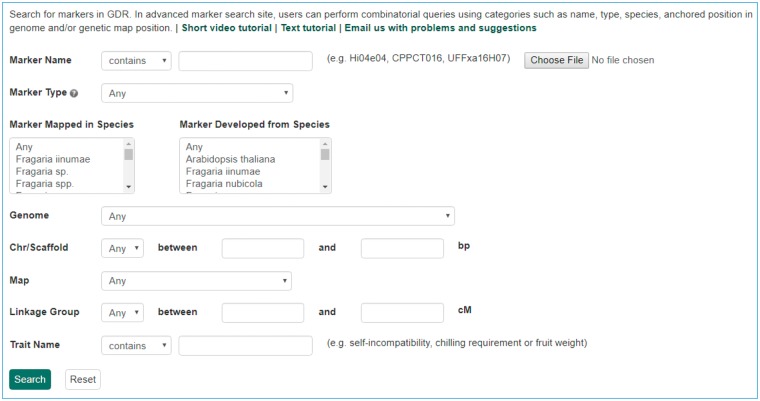
Search page for markers in GDR. Users can search markers by name, type, species, aligned genome positions, genetic map positions and associated trait names.

**Figure 8.**
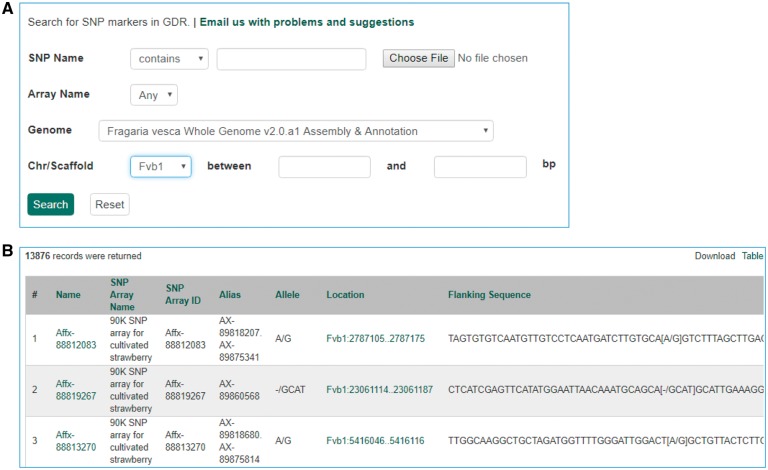
Search page for SNP markers in GDR. (**A**) SNP marker search page where users can search SNPs by name, SNP array name and anchored genome position. (**B**) The returned search results include name, SNP array name, SNP array ID, aliases, alleles, genome location and flanking sequences.

**Figure 9.**
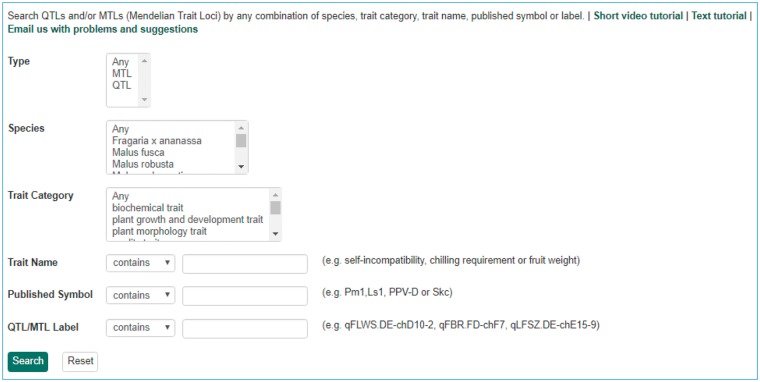
Search page for QTL in GDR. Users can search QTL or MTL by type, species, trait category, trait name, published symbol and/or label.

**Figure 10.**
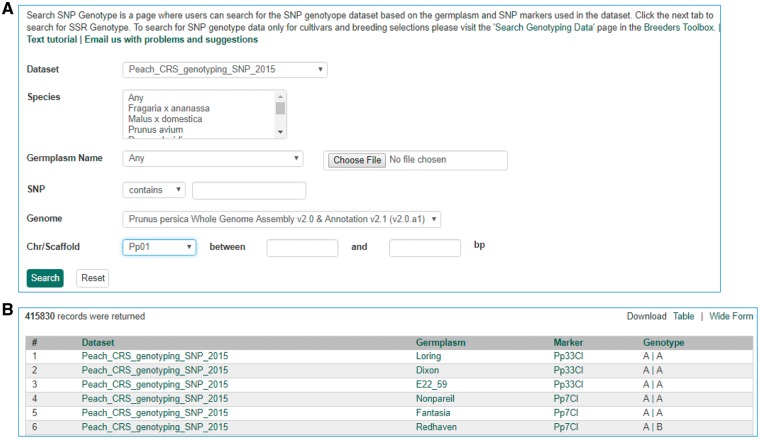
Search page for SNP genotype. (**A**) Users can search SNP genotype by dataset name, species, germplasm name, SNP name and/or genome location of the SNP. (**B**) The returned search results show dataset name, germplasm, marker name and genotype.

**Figure 11.**
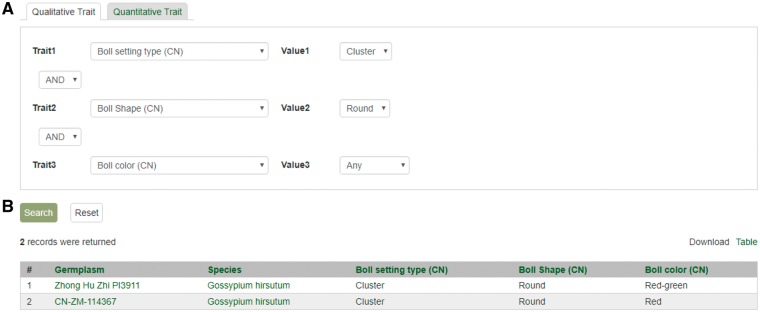
Search page for phenotype in CottonGEN. (**A**) There are two tabs, one for quantitative trait and the other for qualitative traits. In each page, users can choose up to three trait names and the range of phenotypic values to obtain germplasm that has the specified phenotype. (**B**) The returned search results show dataset name, germplasm, species and the trait values for the traits chosen.

The Mainlab Chado Search module provides comprehensive search pages for various types of data: genes, sequences, markers, germplasm, germplasm images, QTL, haplotype blocks, genetic maps, SSR genotypes, SNP genotypes and phenotypes. Once installed, each search page can be enabled or disabled in the admin page (Figure 5). These search pages allow end-users to find data using a series of data filters and then download the results in popular file formats such as CSV and FASTA as appropriate. Figure 6 shows one example of a search page for genes and transcripts available on GDR. Users can search genes and transcripts by genus, species, dataset, aligned genome location, name and keyword (e.g. function or imputed function). The results are returned in a table with gene or transcript name, organism, type, dataset and genome location. Users can download the table in a CSV file, compatible with Excel or the sequences in a FASTA file. Figure 7 shows the marker search page available on GDR. Users can search markers by name, type, species, aligned genome positions, genetic map positions and associated trait names (Figure 7). There is a separate search page for SNP markers where users can search SNP markers by name, SNP array name, anchored genome position (Figure 8A) and the returned search results include name, aliases, array name, alleles, genome location and flanking sequences (Figure 8B). There are also various other marker search pages where users can search markers by nearby markers, marker source information or mapped positions and obtain only those relevant data as a result, implemented in CottonGEN. Figure 9 shows the QTL search page, available on GDR. Users can search QTL or MTL by type, species, trait category, trait name, published symbol and/or label (Figure 9). In the SSR or SNP genotype search page, users can search for genotype data. Figure 10 shows a SNP genotype search page, where users can search SNP genotype by dataset name, species, germplasm name, SNP name and/or genome location of the SNP. The returned search results show dataset name, germplasm, marker name and genotype (Figure 10B). Figure 11 shows the search page for phenotype data in CottonGen. There are two tabs, one for quantitative traits and the other for qualitative traits. In each page, users can choose up to three trait names and the range of phenotypic values to obtain germplasm that has the specified phenotype (Figure 11A). The returned search results show dataset name, germplasm, species and the trait values for the traits chosen (Figure 11B).

The Mainlab Chado Search module uses materialized views to improve the performance of queries. Materialized views are database tables used for aggregating data that would otherwise be too slow to query from Chado’s highly normalized tables. A materialized view

thus improves the search performance, but also allows the site developer to use the search module when data may be stored in slightly different ways in Chado. The site developer would need to modify the query that populates the view to match their data storage strategy. The customization of materialized views is performed using an existing Tripal interface. Instructions for creating a new custom search page are provided to the site developers in the README document that accompanies the module. This module and user documentation are available for download at https://github.com/tripal/chado_search/releases/latest.

### Mainlab Tripal Chado Data Display module

**Figure 12.**
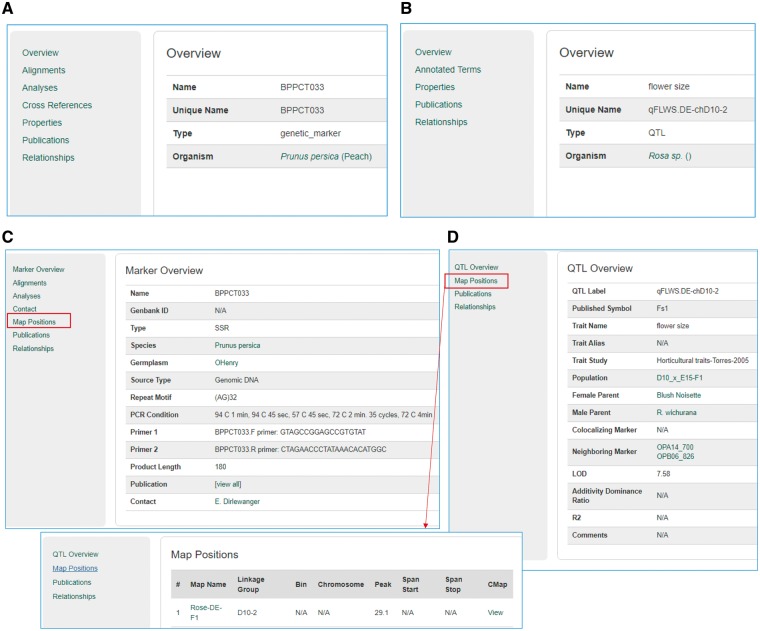
Sample pages from the Tripal core module and the Mainlab Tripal Chado Data Display module. (**A**) A marker page using the feature template from the Tripal core module. (**B**) A QTL page using the feature template from the Tripal core module. (**C**) A marker page using the marker template from the custom module. (**D**) A QTL page using the QTL template from the custom module.

The Mainlab Tripal Chado Data Display module contains a set of Drupal template files that customize any page on a Drupal site including those provided by Drupal and Tripal. By default, Tripal provides template files for many data type pages. However, the template files provided by the Mainlab Tripal Chado Data Display module provides improved displays for some existing data types already supported by Tripal as well as other data types not directly supported. In total, this module supports improved or novel display of organisms, markers, polymorphisms, alleles, QTL, MTLs, germplasms, maps and projects. These templates provide more informative pages, especially when used in conjunction with the Chado Loader module and support more refined classification of a data type. For example, the Tripal core module comes with a single data template to display any entry from the feature table, regardless of feature type (Figure 12A and B). This custom module provides templates for specific feature types providing better contextual links in the left panel. An example result is shown in Figure 12C and D for marker and QTL, respectively.

**Figure 13.**
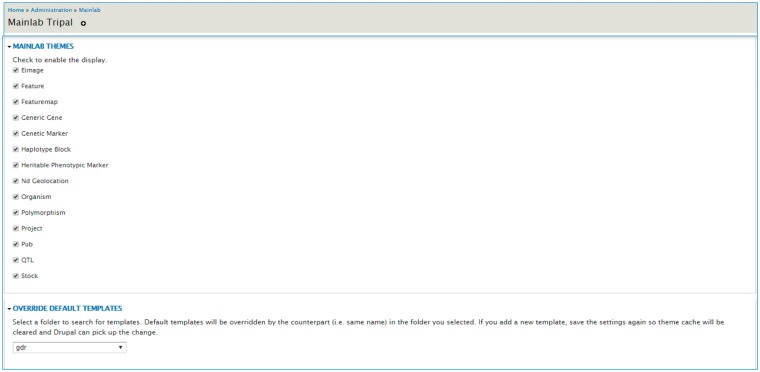
Mainlab Tripal Data Display admin page. Users can enable any templates and choose to over-ride default templates after site-specific modification.

Once installed, site developers can disable any of these templates as appropriate for their database in the admin page (Figure 13). The module supports over-riding built-in templates so site-specific customization is also supported. For customization, the site developer can copy and modify any template provided by this module and enable over-riding default templates in the admin page (Figure 13). Detailed instructions are available in the README document that accompanies the module. This module and user documentation are available for download at https://github.com/tripal/mainlab_tripal/releases/latest.

## Discussion

We reported here our database construction tools for storage, visualization and querying of genomic, genetic and breeding data, Chado Loader, Chado Data Display and Chado Search, which are extension modules of Tripal, a platform for development of online biological databases.

Several data loaders are available in Tripal, such as the GFF3, FASTA, OBO, GAF, NCBI Taxonomy, publication and phylogenetic trees (in Newick format) loaders. Using these loaders, genome data can be loaded relatively easily into Chado and the default Tripal display templates can be used to display these genome data. While these loaders fulfill the needs for many common file formats, the Mainlab modules described here fill an important niche for data that has no standard file format. One other loader of importance is the Tripal bulk loader. This loader allows the site developer to create new loaders for data that are stored in simple tab delimited files. The loader is created using a web interface with no programming required. The Tripal bulk loader is helpful for site developers that have a good understanding of tables in Chado, their foreign key relationship and best practices for storing data. For such users, it can be relatively quick to create a new data loader. However, many of the data templates and loaders provided by the MCL module support data that is often too complex for the bulk loader.

While migrating databases such as GDR and CottonGen to Tripal, the data loaders, search pages and data display templates that handle non-sequence data were converted to be compatible with the Tripal platform. While all of the tools provided by these three modules are related in scope, they are released as three separate modules so that site developers can choose the modules that they need. Within the modules, users can also enable a subset based on the data type they have and also modify them as needed. Communities, labs or individuals that need to build a new online database for genomic, genetic and breeding data can use Tripal and improve its support for these data by downloading and installing the three modules described here. Once installed, these new sites will instantly have data templates for collecting data, loaders to import the templates, as well as improved and customizable search and display pages. The project databases that adopted these modules as well as the Tripal core module include CuttingClass (https://cuttingclass.stowers.org/find/genes) (18), Planosphere (https://planosphere.stowers.org/find/genes) (19) and a private project database SIMRbase (https://genomes.stowers.org).

Future development of these modules includes improvement to data templates for additional metadata, more data templates and loaders as needed, improving search pages, adding more search pages and improving data display templates. The three extension modules are compatible with Chado versions v1.1x, v1.2x and v1.3x and Tripal versions of v2.0 and v2.1. Currently the beta version of Tripal v3.0 is available and the extension modules reported in this paper will be updated when new Tripal versions are released.

## Supplementary Material

Supplementary Data
